# Supplementary material for: Plasma-Induced Changes in the Metabolome Following Vistula Tart Cherry Consumption
Source: Nutrients. 2024 Apr 1;16(7):1023. doi: 10.3390/nu16071023 (PMC11013268; doi:10.3390/nu16071023)
Supplement: Supplementary file 1 [file nutrients-16-01023-s001.zip › Supplementary Documents - v2.docx]

**Supplementary Documents**

**Table S1:** Gradients and time at separation.

| Time (min) | % A | % B |
| --- | --- | --- |
| 0.00 | 94 | 6 |
| 5.00 | 70 | 30 |
| 10.00 | 50 | 50 |
| 12.50 | 40 | 60 |
| 15.00 | 40 | 60 |
| 16.00 | 94 | 6 |
| 25.00 | 94 | 6 |

**Figure S1:** Melatonin Calibration Curve Response


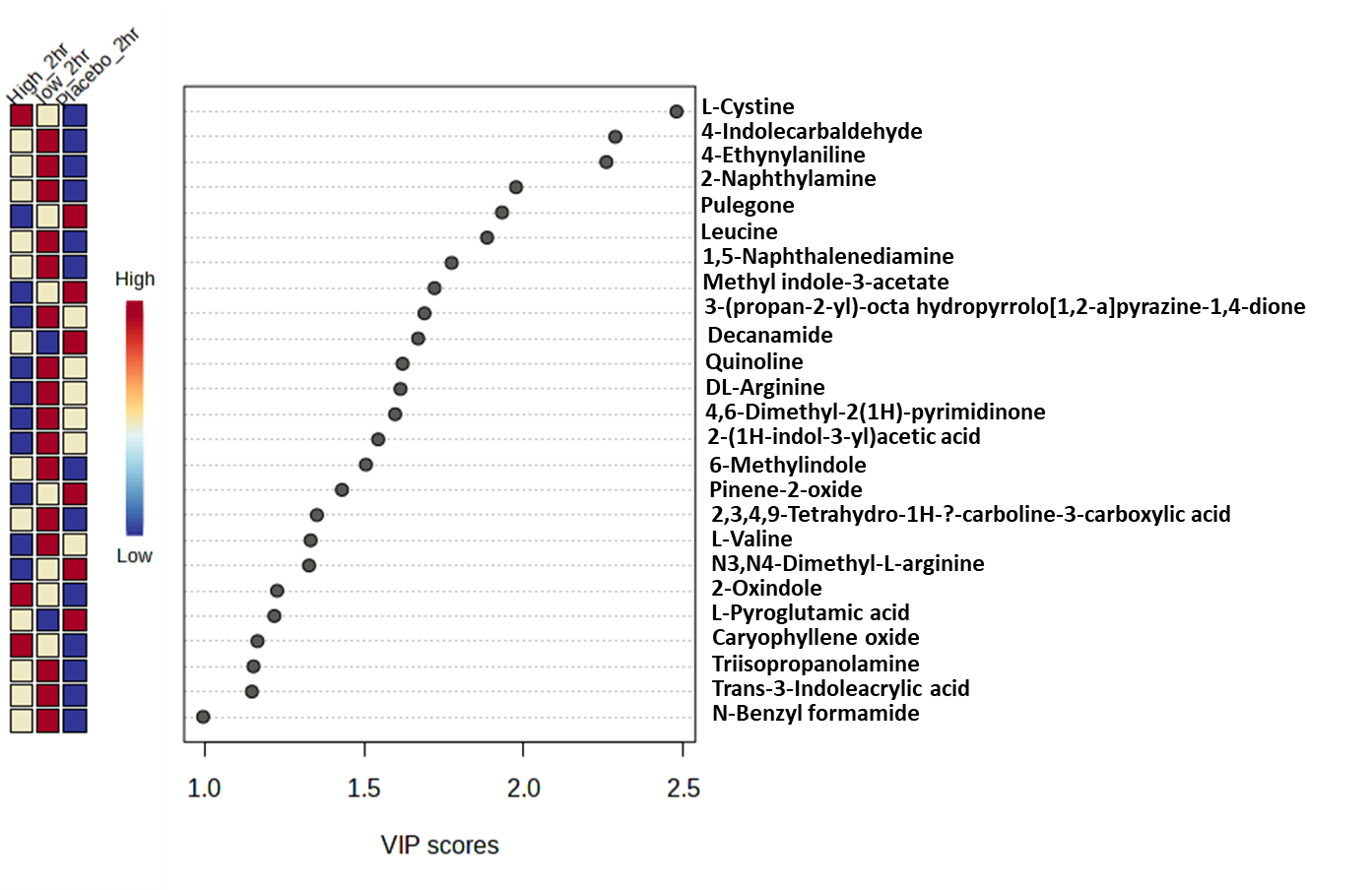


**Figure S2**: PLS-DA ranking of the dysregulate metabolites and VIP scores at 2 h (VIP score x > 1 is statistically significant).


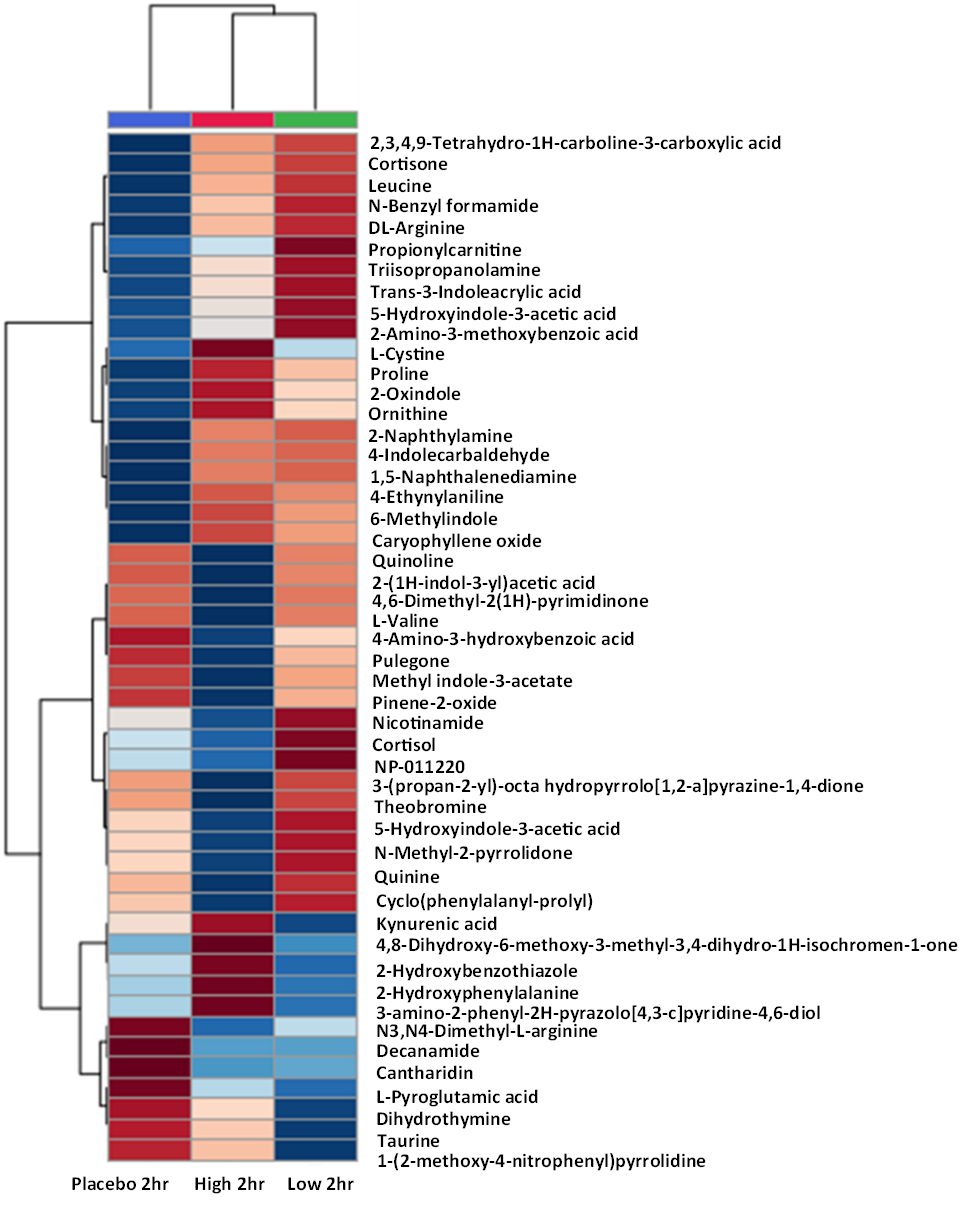


**Figure S3**: Class average heatmap of the top 40 metabolites (ID using PLS-DA at 2 h).


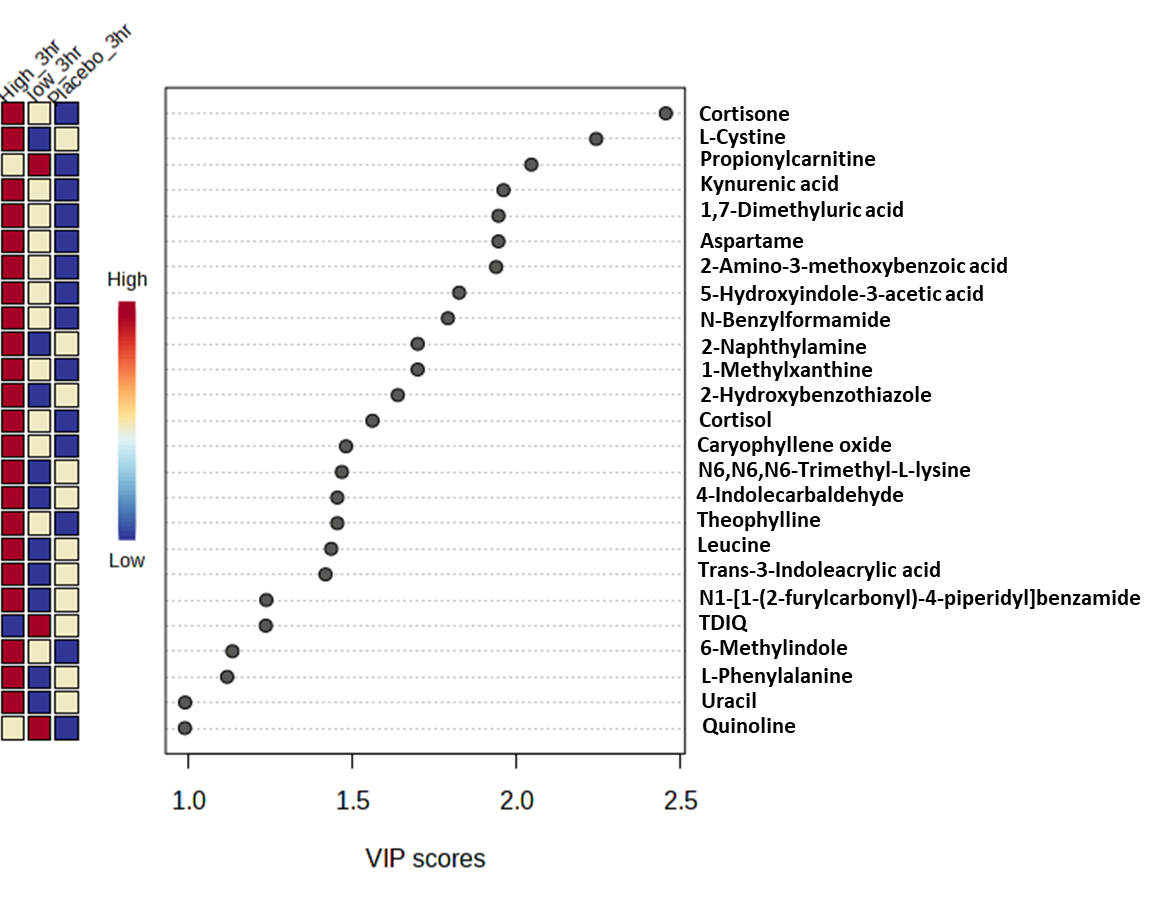


**Figure S4**: PLS-DA ranking of the dysregulate metabolites and VIP scores at 3 h (VIP score x > 1 is statistically significant).


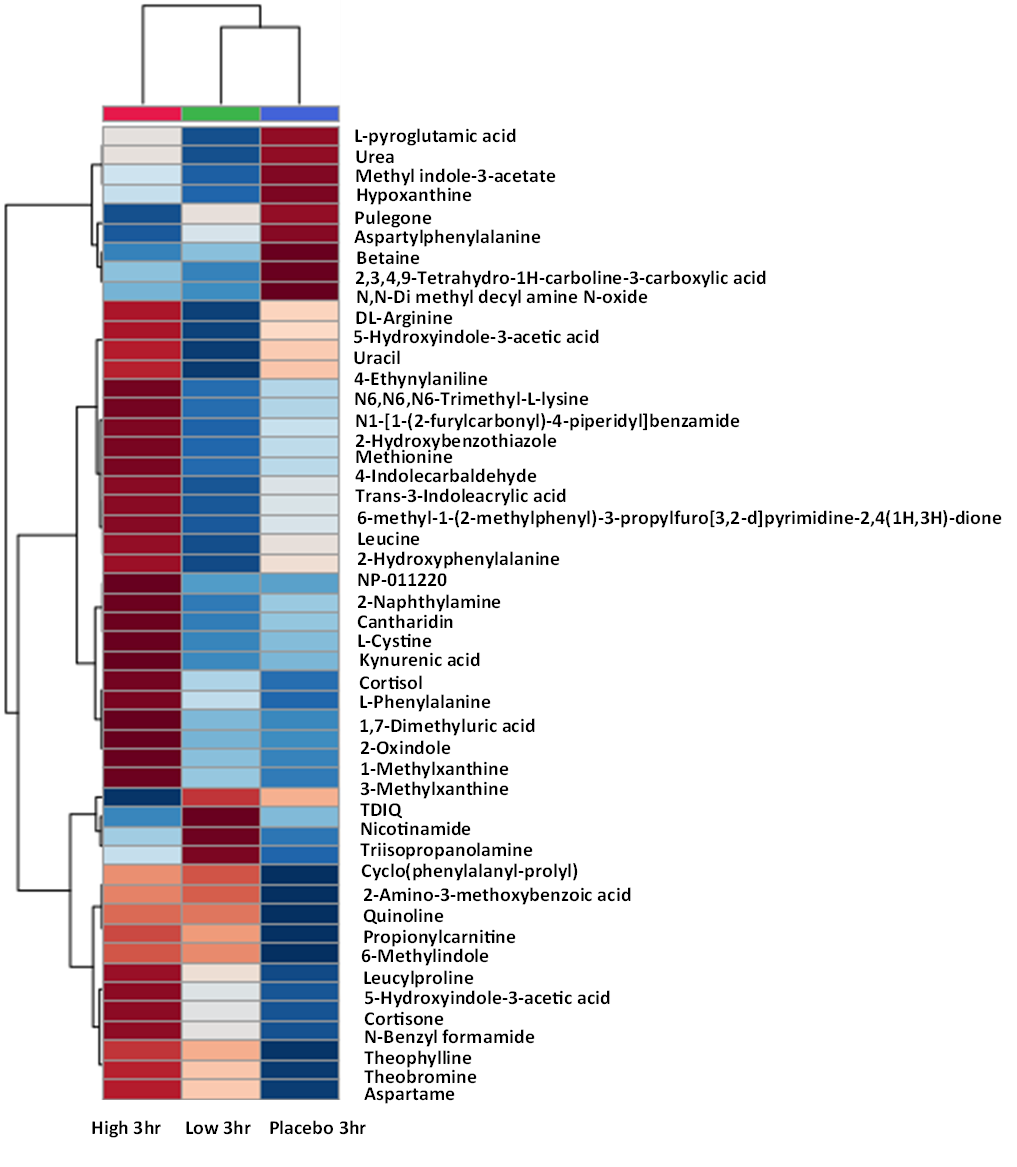


**Figure S5**: Class average heatmap of the top 40 metabolites (ID using PLS-DA at 3 h).


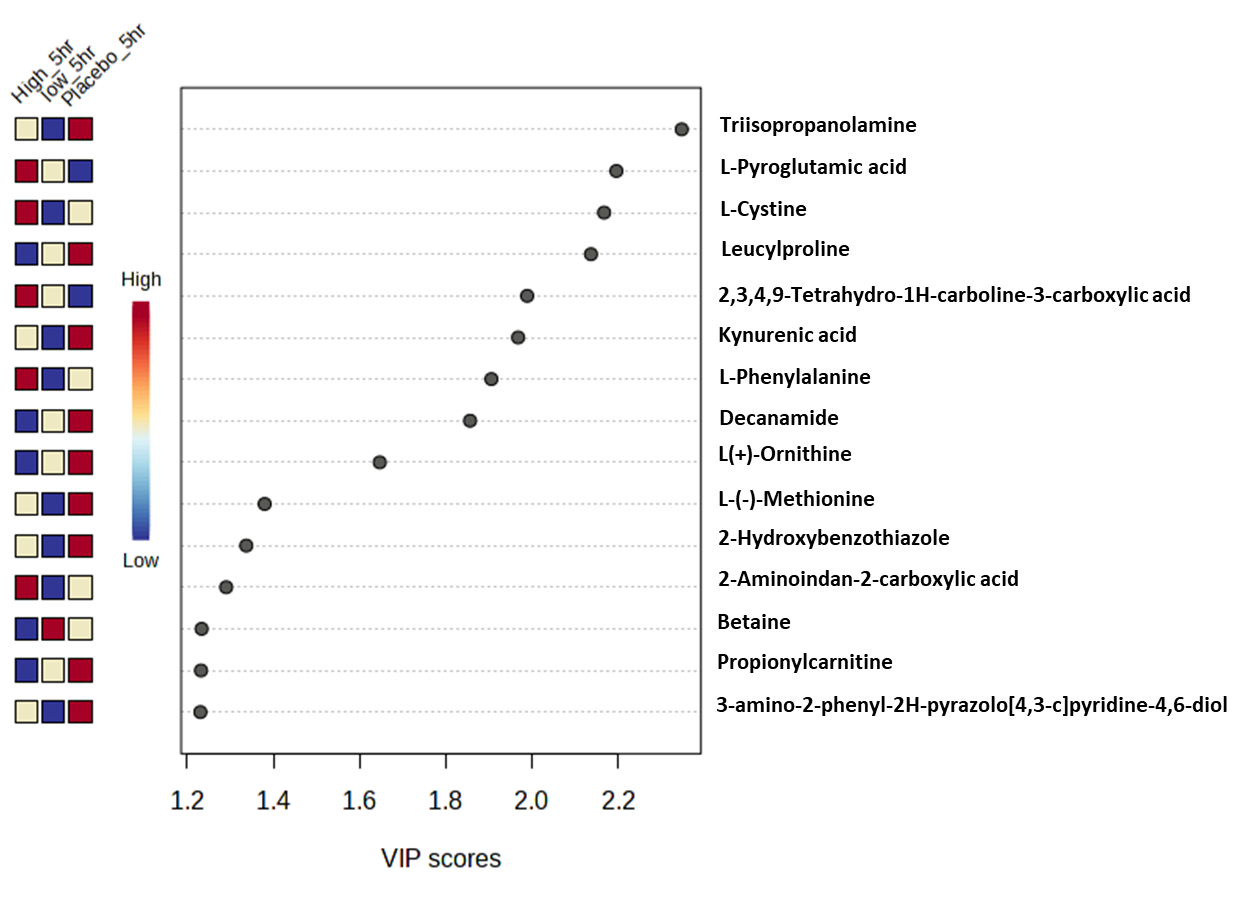


**Figure S6**: PLS-DA ranking of the dysregulate metabolites and VIP scores at 5 h (VIP score x > 1 is statistically significant).


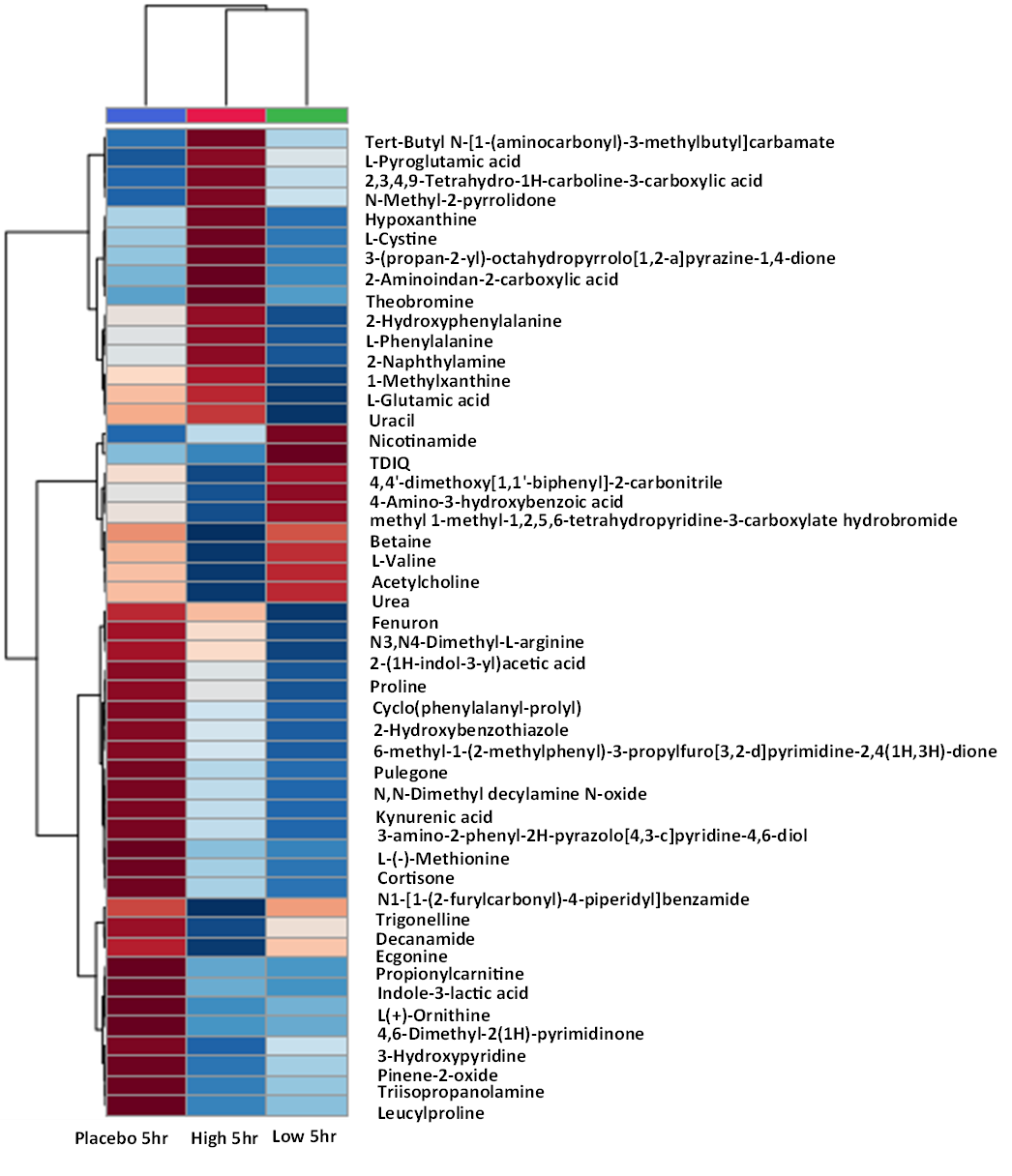


**Figure S7**: Class average heatmap of the top 40 metabolites (ID using PLS-DA at 5 h).


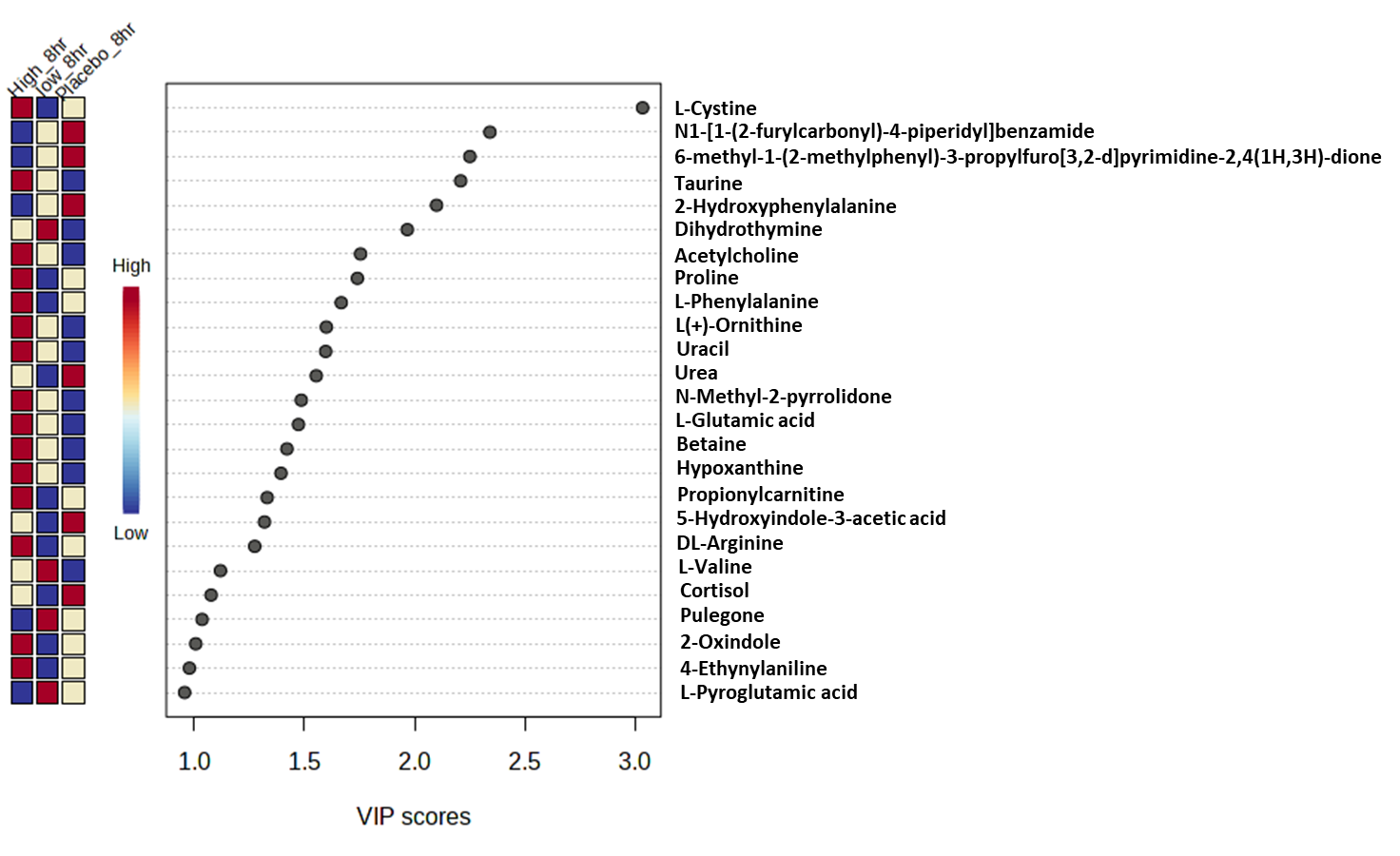


**Figure S8**: PLS-DA ranking of the dysregulate metabolites and VIP scores at 8 h (VIP score x > 1 is statistically significant).


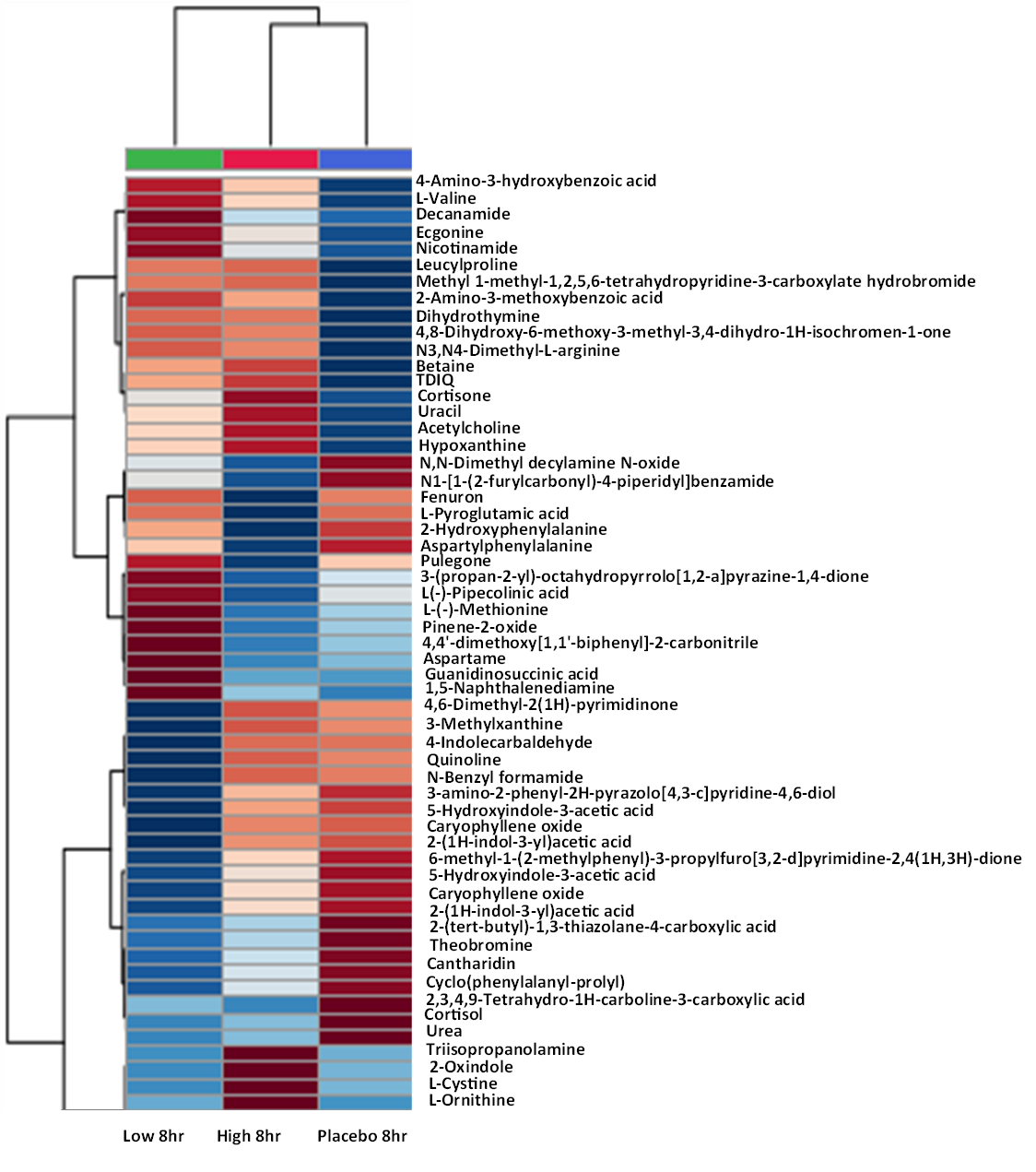


**Figure S9**: Class average heatmap of the top 40 metabolites (ID using PLS-DA at 8 h).
